# Tripartite Chromatin Localization of Budding Yeast Shugoshin Involves Higher-Ordered Architecture of Mitotic Chromosomes

**DOI:** 10.1534/g3.118.200522

**Published:** 2018-07-12

**Authors:** Xiexiong Deng, Min-Hao Kuo

**Affiliations:** Department of Biochemistry and Molecular Biology, Michigan State University. East Lansing, MI 48824

**Keywords:** *Saccharomyces cerevisiae*, spindle assembly checkpoint, Shugoshin, histone H3, tension sensing

## Abstract

The spindle assembly checkpoint (SAC) is key to faithful segregation of chromosomes. One requirement that satisfies SAC is appropriate tension between sister chromatids at the metaphase-anaphase juncture. Proper tension generated by poleward pulling of mitotic spindles signals biorientation of the underlying chromosome. In the budding yeast, the tension status is monitored by the conserved Shugoshin protein, Sgo1p, and the tension sensing motif (TSM) of histone H3. ChIP-seq reveals a unique TSM-dependent, tripartite domain of Sgo1p in each mitotic chromosome. This domain consists of one centromeric and two flanking peaks 3 – 4 kb away, present exclusively in mitosis. Strikingly, this trident motif coincides with cohesin localization, but only at the centromere and the two immediate adjacent loci, despite that cohesin is enriched at numerous regions throughout mitotic chromosomes. Chromosome conformation capture assays reveal apparent looping at the centromeric and pericentric regions. The TSM-Sgo1p-cohesin triad is therefore at the center stage of higher-ordered chromatin architecture for error-free segregation.

Equal partition of the duplicated chromosomes is crucial for genome integrity and species perpetuation. Aneuploidy resulting from erroneous segregation causes developmental defects and tumorigenesis (Ricke *et al.* 2008). The spindle assembly checkpoint (SAC) is a failsafe for faithful segregation. The SAC registers the kinetochore-microtubule attachment and the tension between sister chromatids (Pinsky and Biggins 2005). The tension generated by poleward pulling of the spindles signals bipolar attachment, after which cells irreversibly initiates events leading to the onset of anaphase.

In *Saccharomyces cerevisiae*, each kinetochore attaches to a single microtubule spindle emanating from the spindle pole bodies (Cleveland *et al.* 2003). To the two sister kinetochores, three types of attachment may occur: monotelic, syntelic and amphitelic (Pinsky and Biggins 2005). While the amphitelic attachment signals biorientation, monotelic and syntelic attachment errors have to be corrected before anaphase onset. Monotelic attachment refers to the situation when only one of the two sister kinetochores is attached to the microtubule. The presence of an unoccupied kinetochore triggers the formation of the Mitotic Checkpoint Complex (MCC) (Brady and Hardwick 2000) that halts cell cycle progression by trapping Cdc20p, the E3 ligase subunit of Anaphase Promoting Complex (APC). In syntelic attachment, both sister kinetochores are occupied by spindles, but these two spindles originate from the same spindle pole body. Even though the attachment requirement is met, there may be no tension between syntelic sister chromatids as they are pulled toward the same pole. Left uncorrected, monotelic and syntelic attachment results in aneuploidy.

In what form tension is perceived by the mitotic machinery remains elusive. In prometaphase, transient sister chromatid separation without cohesin proteolysis is caused by kinetochore-microtubule attachment (He *et al.* 2000; He *et al.* 2001). Conformational changes of centromeric chromatin (DNA, nucleosomal arrays, and selective proteins) thus are suggested to be the “tensiometer” or “spring” that reflects the tension status (Salmon and Bloom 2017). Among these candidates, Shugoshin proteins are of particular interest. Shugoshin is a family of conserved proteins playing critical roles in ensuring appropriate chromatid cohesion during cell division (Marston 2015). The budding yeast Shugoshin, Sgo1p, was first identified as a protector of meiotic cohesin against precocious cleavage (Kitajima *et al.* 2004), and later found to be also crucial for cells to activate the SAC in coping with tensionless conditions in mitosis (Indjeian *et al.* 2005). Expressed in S and M phases of the cell cycle (Indjeian *et al.* 2005; Eshleman and Morgan 2014), Sgo1p is localized to centromeres and pericentromere (Kiburz *et al.* 2005; Fernius and Hardwick 2007; Kiburz *et al.* 2008) without stashing a significant extrachromosomal pool (Buehl *et al.* 2018). Shugoshin is recruited to centromeres by binding to histone H2A phosphorylated by the Bub1 kinase (Kawashima *et al.* 2010; Liu *et al.* 2013a). The centromeric recruitment of budding yeast Sgo1p may also involve the interaction with the centromere-specific histone H3 variant Cse4p (Mishra *et al.* 2017). In human mitotic cells, Sgo1 recruited to the outer kinetochore nucleosomes is then driven by RNA polymerase II to the inner centromere where it is retained by cohesin (Liu *et al.* 2015). Besides cohesin, the fission yeast meiosis-specific Shugoshin Sgo1 interacts with the heterochromatin protein 1 (HP1) homolog Swi6 that docks on the heterochromatic mark H3K9me3 in pericentromere (Yamagishi *et al.* 2008; Isaac *et al.* 2017). Unlike other eukaryotes where heterochromatic marks decorate pericentromere to create a footing for Shugoshin, budding yeast lacks such heterochromatic features in the region immediately next to centromeres (Cleveland *et al.* 2003). The geographic pericentromere recruitment of Sgo1p in budding yeast, instead, is accomplished by the association with the tension sensing motif (TSM) of histone H3 in pericentric regions (Luo *et al.* 2010; Luo *et al.* 2016). TSM (^42^KPGT) is a conserved β-turn that connects the flexible N’ tail to the rigid histonefold domain of H3 (White *et al.* 2001). Mutations at K43, G44, or T45 diminish the pericentric localization of Sgo1p and obliterate the cellular response to defects in tension. Restoring pericentric association of Sgo1p by overexpression, via Sgo1p-bromodomain fusion (Luo *et al.* 2010), or by mutating the inhibitory residues K14 or K23 of the H3 tail (Buehl *et al.* 2018) rescues the mitotic defects of these TSM mutations, thus manifesting the pivotal role of Sgo1p retention at the pericentromere. Sgo1p is removed from chromatin after tension is built up in the metaphase (Nerusheva *et al.* 2014). The inverse correlation between Sgo1p retention and amphitelic attachment suggests that Sgo1p is an integral part of the gauge by which cells use to monitor the tension status.

In addition to the TSM, another factor important for targeting Sgo1p to the pericentromere is the cohesin complex. Mutations that impair cohesin loading ablate pericentric localization of Sgo1p, while leaving the centromeric Sgo1p largely unaffected (Kiburz *et al.* 2005). A similar contribution of cohesin to Sgo1 localization has been observed in human systems as well (Liu *et al.* 2015). Cohesin performs its tension sensing-related function by facilitating the formation of the “C” loop of chromatin near the centromeres in mitosis (Yeh *et al.* 2008; Stephens *et al.* 2011). Direct interaction between cohesin and the human Sgo1 has been reported (Liu *et al.* 2013b). The triad of Sgo1, H3 TSM, and cohesin thus likely constitute the core of the tension sensing device. The present work presents evidence for a cohesin- and TSM-dependent tripartite chromatin localization domain of Sgo1p that also involves high-ordered chromatin architecture.

## Materials and Methods

### Yeast strains and plasmid constructs

The yeast strains, plasmids, and primers used in this work are listed in Supplemental Tables 1 to 3.

To study the genome wide localization of Sgo1p, the 6HA epitope-tagged Sgo1p strains, yJL345 (H3WT) and yJL346 (H3G44S) were constructed as previous described (Luo *et al.* 2010). The Sgo1p overexpression strains, yJL322 (H3WT) and yJL324 (H3G44S) were generated by transforming pJL51 (a *URA3* plasmid with p*ADH1*-3HA-*SGO1*-t*ADH1*) into yMK1361 and yJL170, whose endogenous *SGO1* gene was deleted using *TRP1* marker. To ChIP Mcd1p, a 13Myc tag was introduced to the C terminus of *MCD1* locus in yJL347 using pFA6a-13Myc-His3MX6 plasmid as described (Petracek and Longtine 2002). The resultant strain yXD225 was transformed with either pMK439H3WT or pMK439H3G44S (a *LEU2* plasmid bearing all four core histone genes) and followed by 5-FOA selection to select against pMK440 (a *URA3* plasmid bearing all four core histone genes) containing cells, generating yXD233 (H3WT) and yXD234 (H3G44S). *BAR1* was deleted in yXD233 and yXD234 to yield yXD237 and yXD238 respectively, using homologous recombination approach with *URA3* marker. Another version of *bar1* deletion was made in yXD233 to yield yXD282, using *URA3* recycling approach as described previously (Akada *et al.* 2002). An adapted *URA3* recycling method was used to replace the CAR sequence between *RAD57* and *MAF1* with *GAL1* promoter. There were 4 steps PCR to attain the recombinant fragment. Step 1, primers oXD236 and oXD237 were used to amplify 3′ end of *RAD57* from genomic DNA. Step 2, amplified *pGAL1* from plasmid pFA6a-*TRP1-pGAL1-3HA* with primers oXD252 and oXD253. Step 3, PCR the *URA3* from plasmid pMK440 using primers oXD254, oXD255 and oXD240. Step 4, combined PCR products from the previous three steps and used primers oXD236 and oXD240 to amplify the final fragment. The resultant DNA was transformed into yXD282 to attain Ura^+^ transformant, which was then subjected to 5-FOA selection to generate yXD286.

### Yeast methods

Yeast growth media, conditions, and transformation were based on standard procedures (Sherman 1991). When appropriate, 5% casamino acids (CAA) were used to substitute for synthetic amino acid mixtures as selective medium for uracil, tryptophan, or adenine prototroph. Yeast transformation was done with the lithium acetate method (Gietz *et al.* 1992).

### ChIP-qPCR and ChIP-seq

ChIP was conducted as previously described (Kuo and Allis 1999; Luo *et al.* 2010). To quantify the ChIP results, ChIP DNAs were analyzed with quantitative PCR using primers from Table S3. The libraries of Sgo1p ChIP-seq were prepared as described previously (Ford *et al.* 2014). 10 ng of ChIP DNA was used for each library preparation. Size selection of libraries was 300-500 bp. Libraries passed quality control were then subjected to Illumina HiSeq 2500 to get 50 bp single-end reads. Reads were mapped to *S. cerevisiae* genome (Saccer 3.0) by Bowtie2 (version 2.2.6) using -m 1 setting for unique matching reads. BEDgraph files of each ChIP-seq experiments were generated by HOMER (version 4.7.2) and were visualized by Intergrative Genomics Viewer (Broad Institute). Read analysis across centromeres was done by using code of Cen-boxplot_100kb.pl adapted from Verzijlbergen *et al.* (2014). All ChIP-seq data in this study are available at the Gene Expression Omnibus with accession number GSE110953.

### Chromosome Conformation Capture, 3C

3C was performed in 100 OD_600_ cells of G1 or G2M arrest cells as previously described (Belton and Dekker 2015). Instead of using mortar and pestle to lyses cells, 50 U/mL lyticase was used to digest the cell wall for 25 min at room temperature. Primers are designed around 50 bp upstream of the targeted *Eco*R I sites. The digestion efficiency of each libraries was evaluated by qPCR. Samples with at least 70% digestion were carried on for following assay. PCR products were resolved by 9% PAGE and stained by ethidium bromide. The intensity of band was analyzed by NIH ImageJ.

### Data availability

Strains and plasmids are available upon request. Supplemental material available at Figshare: https://doi.org/10.25387/g3.6227180.

## Results

### Sgo1p displays unique tripartite localization in each mitotic chromosome

**Figure 1:**
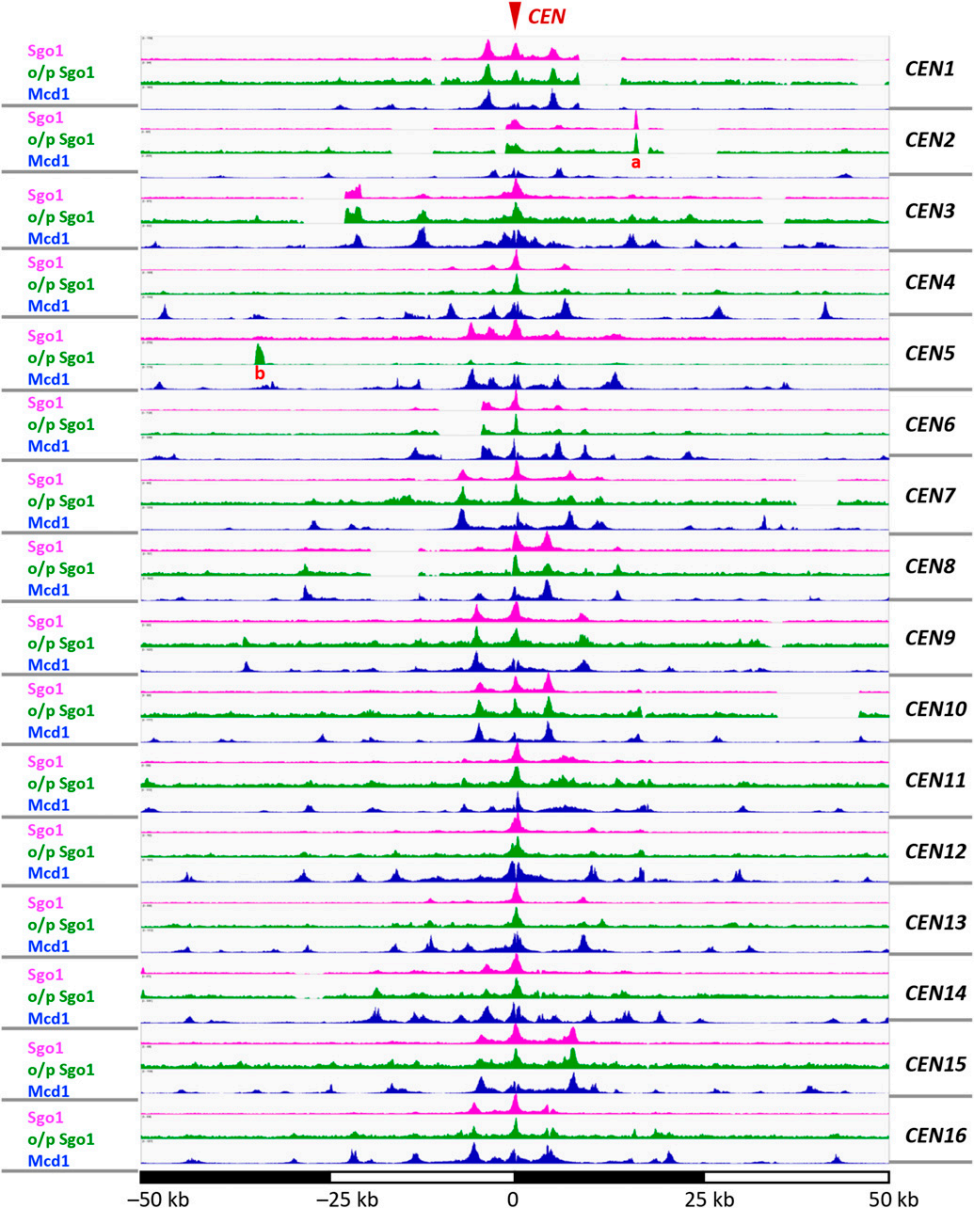
Sgo1p is recruited to centromeres and pericentromere to form a tripartite localization domain on each mitotic chromosome. The 100-kb region centering on the centromere of all 16 chromosome is aligned. Sgo1p expressed from its native locus (magenta), or from a multi-copy episomal plasmid (green) are compared with the Mcd1p distribution (dataset from Verzijlbergen *et al.*, 2014). The two peaks labeled “a” and “b” close to *CEN2* and *CEN5* correspond to *ARS209* and *URA3* respectively. These loci were from two plasmids in the strains used for experiments.

**Figure 2:**
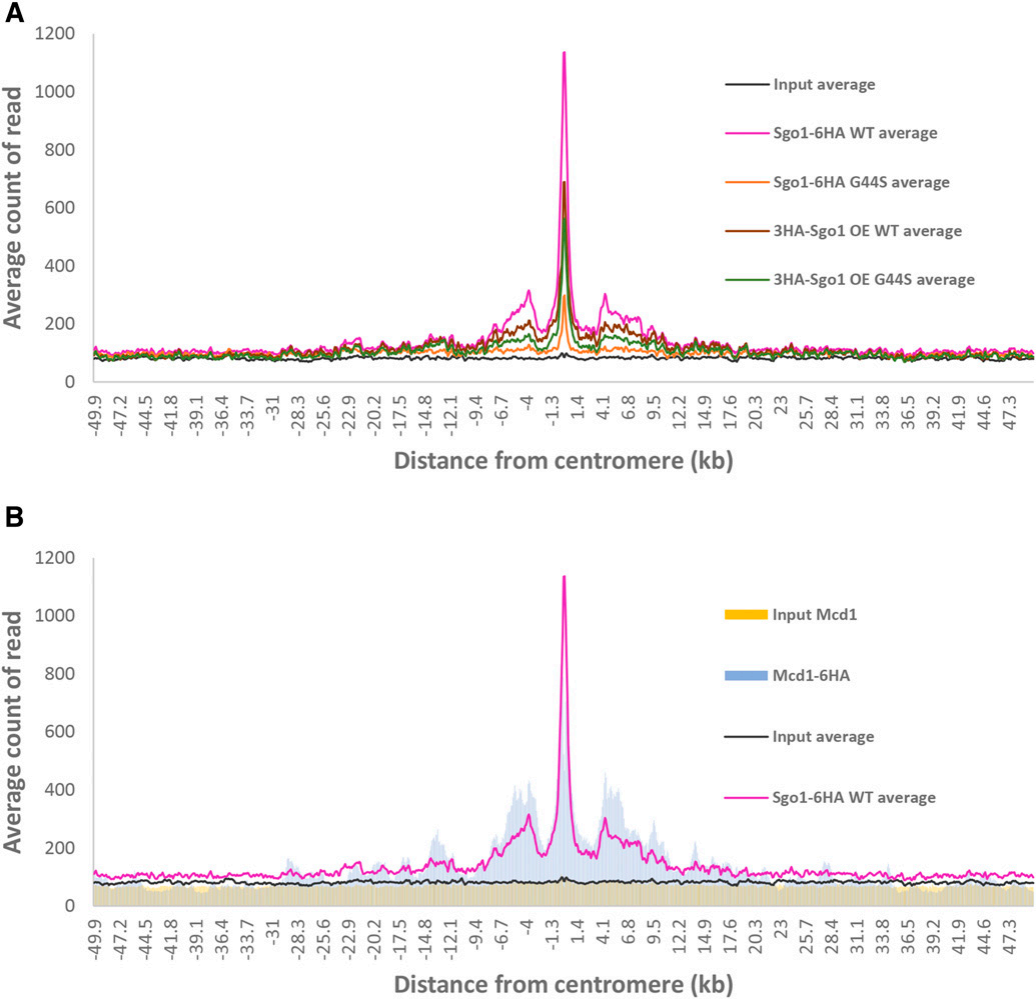
Sgo1p enrichment overlaps with cohesin domains at the centromeres and pericentromere. Average counts (per million reads) plot comparing the distribution of Sgo1p expressed in different backgrounds (panel A) or between Sgo1p and cohesin (panel B). Sgo1-6HA ChIP-seq and 3HA-Sgo1 ChIP-seq data were from three and two biological replicas respectively. The Mcd1p ChIP-seq data were from Verzijlbergen *et al.*, 2014.

Sgo1p is critical for the tension sensing branch of the SAC function in mitosis (Marston 2015). We and others have previously used chromatin immunoprecipitation (ChIP) to demonstrate that Sgo1p is enriched at centromeres and several kb on either side of the centromere in mitosis (Kiburz *et al.* 2005; Fernius and Hardwick 2007; Luo *et al.* 2010; Nerusheva *et al.* 2014). However, the range of the Sgo1p enrichment on each mitotic chromosome has not been carefully delineated. To better understand Sgo1p retention pertaining to its checkpoint function, we used ChIP-seq to map the Sgo1p distribution on mitotic chromosomes at a higher resolution. Cells bearing a C-terminally HA-tagged Sgo1p expressed from its native locus were arrested by benomyl for ChIP-seq. At a lower resolution scale, Sgo1p is detectable in one area per mitotic chromosome (Figure S1A), consistent with the anticipation of centromeric and pericentric enrichment (Kiburz *et al.* 2005). However, more rigorous inspection revealed that each chromosomal domain of Sgo1p is actually composed of discrete peaks of Sgo1p that form a trident-like structure, not a continuous motif covering several kb of a centromeric and pericentric area (Figure 1). Each of the trident motif consists of a middle centromere (CEN) and typically one pericentromere (PC) peak on each side of the CEN enrichment. Some chromosomes such as I, VIII, and XI, show weaker PC peaks and therefore a less conspicuous tripartite pattern. However, overexpressing Sgo1p from the *ADH1* promoter on a multi-copy plasmid increased the overall IP efficiency (reflected by broader scales) and heightened these peaks (green curves, Figure 1). These observations suggest that the mechanism for Sgo1p retention is conserved, whereas the relative strengths may differ among chromosomes. By aligning all sixteen chromosomes at the centromeres, the average counts plot for Sgo1p enrichment as a function of distance to CEN shows that the average distance between the PC and CEN peaks is approximately 4 kb (Figure 2A, magenta line). Additional outward peaks may be seen in some chromosomes, but the overall peak height drops quickly.

Chromosomal retention of Sgo1p depends critically on the tension sensing motif (TSM) of histone H3 (Luo *et al.* 2010), and the cohesin complex (Verzijlbergen *et al.* 2014). H3 is a ubiquitous component of chromatin, yet it controls the pericentric localization of Sgo1p (Luo *et al.* 2010), despite that no discernible epigenetic marks have been found specifically in budding yeast pericentromere that are relevant to mitotic regulation. Mutations introduced to the tension sensing motif (^42^KGPT^45^) cause defects in detecting and/or responding to tension defects (Luo *et al.* 2016). These mutations diminish the affinity for Sgo1p, a molecular defect that can be suppressed by overproduction of Sgo1p (Luo *et al.* 2010; Luo *et al.* 2016). Indeed, ChIP-seq data show that the overall chromatin association of Sgo1p is significantly reduced in a tension sensing motif mutant, G44S (Figure S1A, orange curve). Overexpressing Sgo1p restored the tripartite chromatin association (green curves, Figure 1, and brown curve, Figure S1A). In addition to re-establishing the original enrichment pattern, a small number of new peaks distal to the CEN/PC peaks were seen. Intriguingly, these still are discrete peaks with clear valleys in between (see, for example, chromosome XVI, Figure 1). The emergence of these new enrichments is consistent with our original model that Sgo1p is recruited to the centromeres and then spills over to the nearby chromatin region (Luo *et al.* 2010). However, the non-continuous nature of Sgo1p distribution suggests the involvement of at least one other factor (see below).

**Figure 3:**
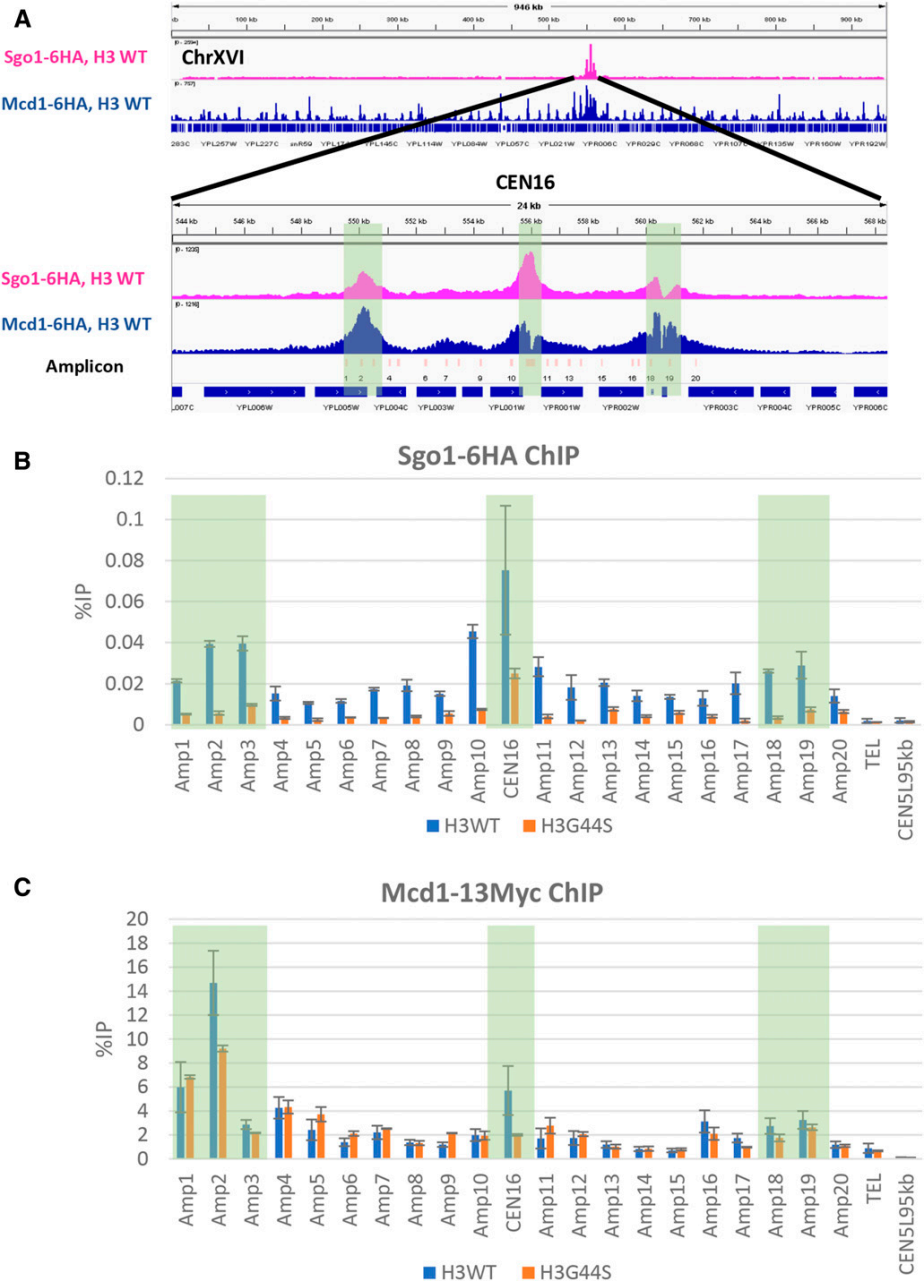
The histone H3 tension sensing motif is essential for pericentric Sgo1p localization but not Mcd1p. A. Distribution of Sgo1p (magenta) and Mcd1p (blue) across chromosome XVI as revealed by ChIP-seq. The centromeric region is blown up to show the detail distribution of these two proteins. PCR amplicons are enumerated and shown in light pink bars below the Mcd1 peaks. The open reading frames and their transcription directions are shown at the bottom. B and C. Quantitative real-time PCR analysis of separate ChIP experiments. Sgo1p-HA and Mcd1p-Myc (both expressed from their native loci) were ChIP’ed from cells bearing the wildtype or a mutant TSM (G44S). The three enrichment sites are marked with shaded boxes. ChIP-qPCR data were from three biological replicas. We repetitively observed that the Mcd1p ChIP signals to be significantly higher than those of Sgo1p (also see Figure 5). This differentiation may result from the choice of the epitope tags (13-Myc *vs.* 6- or 3-HA), or the nature of chromosome association, or both.

While histone H3 and its tension sensing motif are ubiquitously distributed throughout the genome, another Sgo1p recruitment factor, the cohesin complex, localizes at specific loci of chromosomes. Besides centromeres and pericentric regions, the majority of cohesin-associated regions are the intergenic area between two convergent transcription units throughout the genome (Glynn *et al.* 2004; Lengronne *et al.* 2004). By comparing with the chromosomal distribution of Mcd1p (the kleisin subunit of cohesin) (Verzijlbergen *et al.* 2014), we observed that Sgo1p co-localizes with cohesin at and immediately adjacent to centromeres (compare magenta and blue peaks, Figure 1 and Figure 3A). The plot of average count reads (Figure 2B) clearly shows the highly significant co-localization of Sgo1p- and Mcd1p at the centromeric and pericentric region. It is also noteworthy that most additional Sgo1p peaks resulting from overexpression are at the loci where cohesin is also enriched (Figure 1). These results strongly suggest that Sgo1p targets existing cohesin enrichment sites for interaction with the tension sensing motif of histone H3.

In addition to comparing our Sgo1p ChIP-seq data with a published Mcd1p dataset (Verzijlbergen *et al.* 2014), we conducted another set of ChIP assays and used quantitative PCR to examine the localization of Mcd1p and Sgo1p in the same genetic background. To this end, Sgo1p-HA and Mcd1p-Myc expressed from their native loci were subjected to ChIP. DNA products were then examined by quantitative PCR for 21 amplicons that spanned 11 kb of the centromeric region on chromosome XVI, including the three CEN and PC peaks (shaded boxes, Figure 3A top panel). Discrete peaks and valleys are readily visible and show a high degree of overlapping between Sgo1p and Mcd1p with the ChIP-qPCR data. Additional qPCR analysis of chromosome I amplicons equivalent to those of chromosome XVI also verifies the ChIP-seq observations (Figure S2). In addition, parallel ChIP reactions were conducted in the G44S *tsm*^-^ background. While the Sgo1p signals diminish significantly in this region (orange bars, Figure 3C), the Mcd1p-Myc enrichment is not significantly affected, which demonstrates that TSM is required for the retention of Sgo1p, not Mcd1p, at pericentromere.

**Figure 4:**
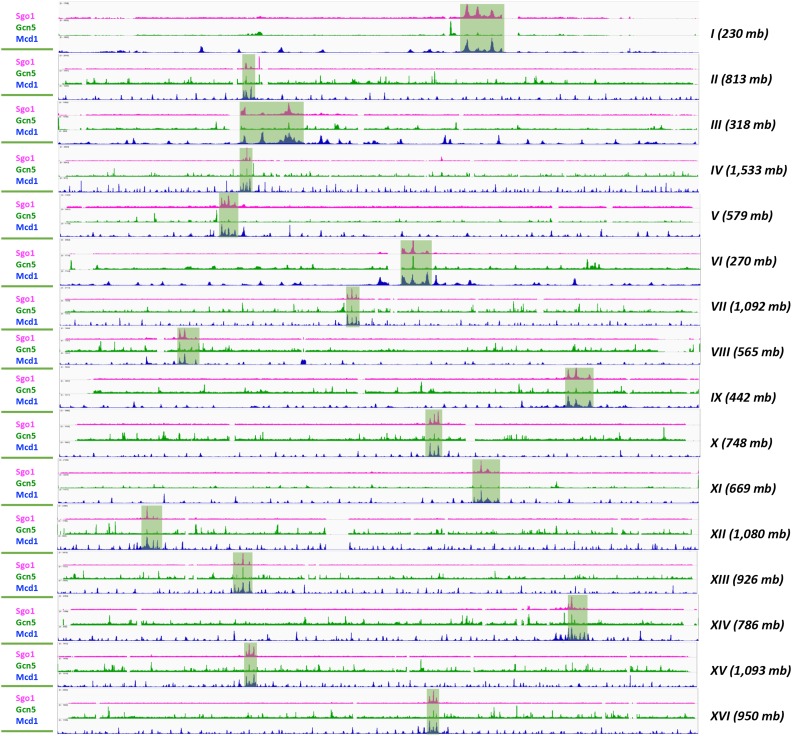
Gcn5p is enriched in centromeres but shows no overlap with cohesin elsewhere. Genome-wide distribution of Gcn5p is compared with that of Sgop1 (magenta) and Mcd1p (blue). The trident Sgo1p localization domain in each chromosome is marked with the shaded boxes.

The exceptional selectivity of Sgo1p for a subset of cohesin localization motifs prompted us to compare its genome-wide distribution to that of Gcn5p in mitotic chromosomes. Gcn5p is a critical transcription regulatory histone acetyltransferase. In mitosis, Gcn5p negatively regulates the tension sensing motif (Luo *et al.* 2016), and is important for maintaining the normal centromere chromatin structure (Vernarecci *et al.* 2008). Consistently, Gcn5p is present at mitotic centromeres (Luo *et al.* 2016). To see whether Gcn5p exhibits a mitotic chromosome localization pattern similar to that of Sgo1p, ChIP-seq was conducted on a Myc-tagged Gcn5p. The results show that, while Gcn5p is found enriched at all centromeres, its pericentric presence is practically negligible (shaded boxes showing CEN/PC peaks of Sgo1p, Figure 4). Importantly, throughout the genome, there is very little overlapping between Gcn5p and Mcd1p enrichment. This is not unexpected for Gcn5p is recruited to the 5′ region of many genes for transcriptional regulation, but Mcd1p and the rest of the cohesin complex are enriched at the intergenic region of convergent genes. There appears to be an enrichment of Gcn5p at RNA polymerase III-controlled targets, such as tRNA genes. These ChIP-seq results are consistent with the canonical roles of Gcn5p in transcription (Venters *et al.* 2011), although we do not exclude the possibility that at least part of the mitotic distribution pattern of Gcn5p might be for chromatin metabolism during mitosis. Together, ChIP-seq data presented above reveal unique association between Sgo1p and Mcd1p at and near the centromeres. However, this connection does not apply to the recruitment of Gcn5p, indicating a specific functional interplay between Sgo1p and the cohesin complex.

The cohesin complex is required for chromatin association of Sgo1p in both budding yeast and human (Kiburz *et al.*, 2005, Liu *et al.*, 2015). To further confirm that the highly specific centromeric and pericentric localization of Sgo1p requires the local cohesin populations, we took two approaches. First, we deleted *IML3* that encodes a subunit of the Ctf19 kinetochore subcomplex. ChIP and quantitative PCR analysis shows that this manipulation disrupts only the pericentric, but not chromosome arm recruitment of cohesin (Kiburz *et al.* 2005) (Figure S3A). As predicted, the pericentric Sgo1p enrichment in chromosome XVI is completely lost in *iml3∆* cells (Figure S3B). In the second approach, we targeted a specific cohesin associated region (CAR) on chromosome IV for inducible disruption. Active transcription can dislodge cohesin enrichment (Glynn *et al.*, 2004). Accordingly, we replaced the pericentric CAR between YDR004W and YDR005C to a galactose-inducible promoter *GAL1* (*pGAL1*, Figure S4). Changing from a non-inducing (raffinose) to an inducing (galactose) condition caused transcription-driven removal of both cohesin and Sgo1p (Figure S4).

From data presented in Figure 3 and Supplemental Figures 3 and 4, we conclude that the tripartite localization of Sgo1p in each chromosome depends on an intact tension sensing motif and likely is established at pre-existing or concomitantly with cohesin localization domains.

### Pericentric Sgo1p domain formation does not appear to involve intervening valley regions

**Figure 5:**
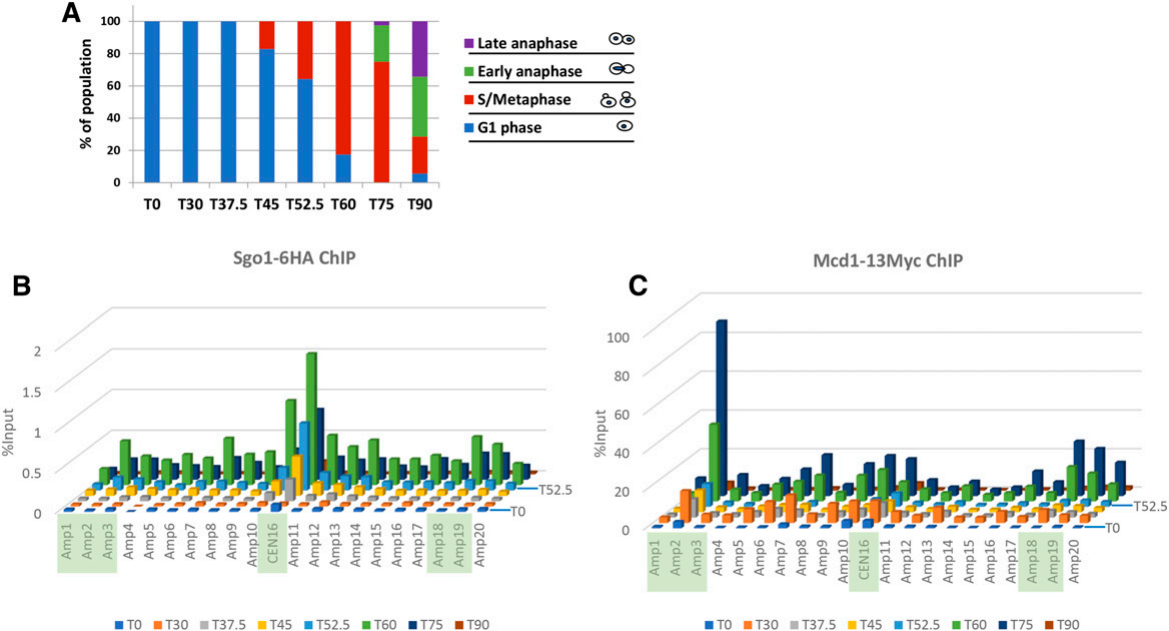
Dynamic recruitment of Sgo1p and cohesin at centromere and pericentromere through cell cycle. A. Budding index of cells collected from the indicated time points. B and C. Sgo1p-HA and Mcd1p-Myc co-expressed in the same cells were examined by ChIP-qPCR. PCR amplicons correspond to *CEN16* and nearby regions. See Figure 3 for positions of these amplicons. In addition to the conspicuous difference in the IP efficiencies between Mcd1p-Myc and Sgo1p-HA (also see Figure 3), we noticed that the ChIP efficiency (%IP) of both Sgo1-6HA and Mcd1-13Myc in cells synchronously progressing through cell cycle was consistently 5- to 10-fold higher than in those benomyl-arrested cells. Results shown in this figure are a representative of two biological replicas. In both cases, the Mcd1p-13Myc exhibited significantly higher IP efficiency.

Sgo1p docks on centromeres via direct association with Bub1p-phosphorylated Ser121 of histone H2A (phos.H2A) within the single centromeric nucleosome (Kawashima *et al.* 2010). Sgo1p also binds the N’ tail of the centromere-specific histone H3 variant, Cse4p (Mishra *et al.* 2017). It is likely that phos.H2A and Cse4p provide the docking site for Sgo1p that nucleates outward spread toward the pericentric regions. The establishment of PC enrichment of Sgo1p may be accomplished by one of two mechanisms. In the rippling mode, a wave of Sgo1p spreads along the nucleosomal path before it stops and accumulates at the first cohesin block. Alternatively, Sgo1p “leaps” directly from centromeres to the PC region where it is retained by the tension sensing motif. In both modes, Sgo1p is underrepresented at the region between the CEN and PC peaks, resulting in the “valleys” seen in the two-dimensional presentation of the ChIP-seq results. These two modes of Sgo1p recruitment can be differentiated by examining the dynamics of CEN and PC peaks emergence when cells progress through mitosis. An intermediate stage where a significant elevation of Sgo1p signals at the valley region before they move outward to generate the final PC peaks would support the rippling mode. To test these two models, we tagged Sgo1p and Mcd1p in the same strain to avoid any variation between cells with different genotypes. Cells expressing Sgo1-6HA and Mcd1-13Myc were arrested in G1 phase by α factor. They were then released into the division cycle before collection at 30, 37.5, 45, 52.5, 60, 75, and 90 min after the release. Budding index revealed the timing of the progression through mitosis during the course of experiments (Figure 5A). ChIP results (Figure 5B and Figure S5) show that Sgo1p was first detectable at *CEN16* 37.5 min after release from G1 arrest, when cells were at the juncture of G1 and S phases. This coincides with the time when Sgo1p expression starts (Indjeian *et al.* 2005). While Sgo1p centromeric abundance continued to rise, the adjacent PC peaks started to surface in the next 7.5 min (amplicons 3, 16, and 21). These signals culminated at T_60’_ (green bars, Figure 5B) and progressively diminished at T_75’_ T_90’_. Between T_60’_ and T_75’_, approximately 20% of cells entered the anaphase (green sector, Figure 5A), indicating that biorientation had been established in this population of cells. The concomitant reduction of Sgo1p signals is in excellent agreement with the tension-dependent removal of Sgo1p from the chromatin (Nerusheva *et al.* 2014).

The kinetics of Mcd1p association with CEN and PC exhibited several important distinctions. First, while Mcd1p signals jumped at T30’, the three subsequent time points (T37.5′, T45’ and T52.5′) saw a reduction of the overall Mcd1p signals, which then climbed up again, and peaked at T75’ before abrupt disappearance by T90’, when the majority of cells passed the metaphase-to-anaphase transition (Figure 5A). The dynamic changes before T60’ probably resulted from transcriptional activities in S and G2 phases. The abrupt increase of Mcd1p signal at T60’ agreed well with the budding index that 80% of the cells were in the metaphase when cohesion of sister chromatids was most critical. Lastly, the highest levels of the Mcd1p abundance were found to be at T75’ before its quick disappearance by T90’, both were 15’ later than Sgo1p. The different kinetics of Sgo1p and Mcd1p dissolution concurs with the anticipated sequence of biorientation, Sgo1p removal, and Mcd1p cleavage that marks anaphase onset.

Similar to results shown in Figure 3, Mcd1p tagged with 13 tandem copies of Myc tag exhibited significantly higher IP efficiency than Sgo1p-6HA. The IP efficiency of Mcd1p-13Myc became even higher in synchronous cultures, suggesting that the cohesin complex plays a major structural role in shaping the pericentric portion of mitotic chromosomes.

One critical observation from results in Figure 5 is that during the formation of the Sgo1p CEN and PC tripartite motif, the two valleys flanking the CEN peak never rose to the levels of PC at any given time. While we cannot formally rule out the possibility that Sgo1p is pushed along the nucleosomal array between CEN and PC peaks at a rate that is significantly faster than the 7.5-minute interval for ChIP assays, given the stochastic nature of cellular physiology even in a synchronized population (see budding index, Figure 5A), the lack of Sgo1p signal at these valley regions favors the notion that Sgo1p spreads from CEN by a “hopping” mechanism to PC, or is recruited simultaneously to CEN and PC to generate the tripartite motif. This conclusion is also consistent with the existence of higher-ordered chromatin architecture near the centromeres as shown below.

### Chromosome conformation capture reveals correlation Between Sgo1p enrichment and chromatin architecture

If Sgo1p targets its pericentric destination immediately after or concomitantly with the centromeric recruitment, it is likely that the PC regions are rendered accessible to Sgo1p whereas the intervening regions are somehow hidden from Sgo1p. Because the interaction between Sgo1p and TSM does not require any posttranslational modification (Luo *et al.* 2010; Luo *et al.* 2016), a non-epigenetic feature may distinguish the PC Sgo1p targets from other areas nearby. We felt that chromatin architecture would be a good candidate that dictates the (in)accessibility of the CEN/PC region to Sgo1p. Compaction of chromatin in mitosis involves condensin and cohesin complexes (Hudson *et al.* 2009; Mehta *et al.* 2013). Both complexes are also shown to be critical for organizing pericentromere in prometaphase (Yeh *et al.* 2008; Nasmyth 2011; Stephens *et al.* 2011). Cohesin facilitates the formation of intrachromosomal centromeric loops for mitotic segregation and resides near the summits of these loops. On the other hand, the condensin complex holds and organizes the bottom of these loops along the spindle axis (Stephens *et al.* 2011). Taking together these models and our results shown above, we suspect that higher-ordered chromosomal architecture, *e.g.*, chromosome looping, might be part of the mechanism underlining the highly selective pericentric localization for Sgo1p.

**Figure 6:**
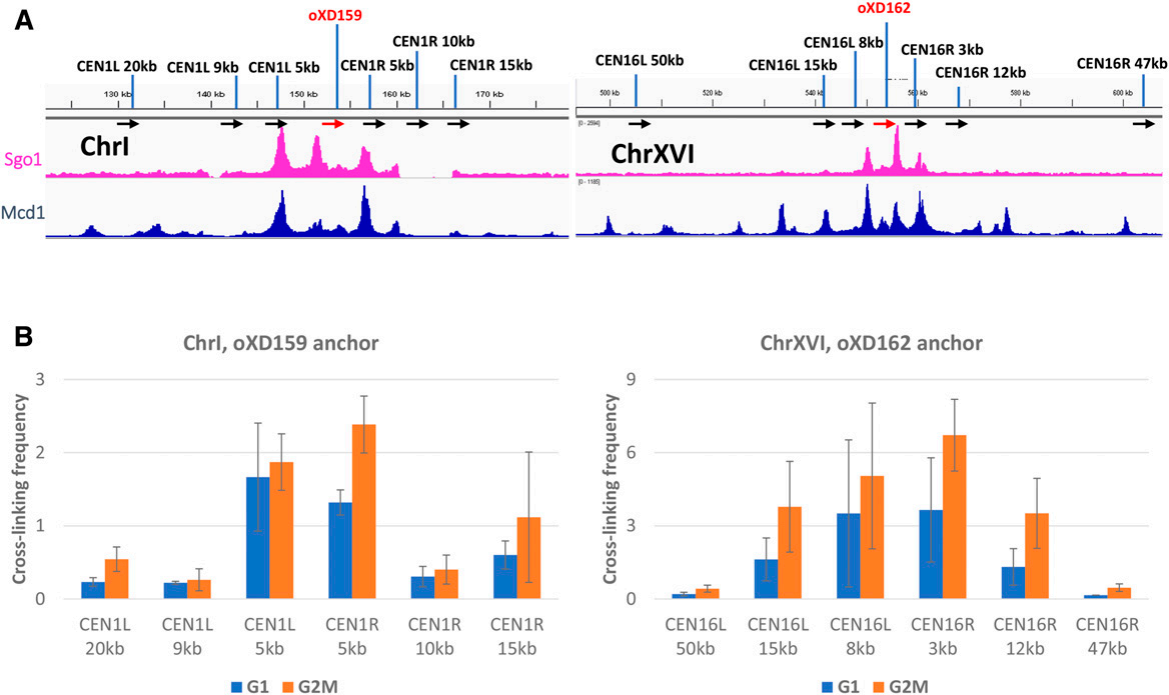
Sgo1p tripartite localization domain is associated with high-ordered chromatin architecture in mitosis. Chromosome conformation capture (3C) assay was used to examine chromatin looping near *CEN1* and *CEN16*. Cells arrested in G1 or G2/M phase were fixed with formaldehyde, and the isolated nuclei treated with *Eco* RI before DNA ligation. An identical amount of final ligated DNA library was amplified by PCR using one of two common anchor primers (oXD159 and oXD162 for chromosomes I and XVI, respectively; red arrows) against different locus-specific primers (black arrows; named for their distance to the centromere, L = left; R = right) 3 – 50 kb away. All primers face toward the same direction. PCR products were resolved by gel electrophoresis and quantified with the NIH Image J software. Shown are the signals relative to the same amplicons without formaldehyde crosslinking. Error bars are standard deviations from three biological replicas.

If Sgo1p recruitment is linked to chromosome looping in mitosis, we predicted that PC and CEN peaks of Sgo1p were spatially near each other owing to the action of such complexes as cohesin and condensin. This hypothesis was tested by chromosome conformation capture (3C) (Dekker *et al.* 2002). Yeast nuclei were harvested from G1 and G2/M arrest and were subjected to *Eco*R I digestion with or without formaldehyde fixation, followed by ligation under a condition that favored intramolecular ligation. The resultant DNA libraries were analyzed by PCR using one of two centromere-proximal anchor primers, oXD159 for *CEN1* and oXD162 for *CEN16*. In each quantitative PCR reaction, these anchor primers were paired with a distal primer that is 3 – 50 kb away (black arrows, Figure 6A). All primers hybridized to the same strand of DNA, hence should not produce any PCR product without the 3C treatment. On the other hand, ligation at the anticipated *Eco*R I sites after formaldehyde fixation would generate templates amplifiable by the anchor and the locus-specific primers. Comparing the intensity of PCR products amplified from samples with or without formaldehyde treatment yielded “crosslinking frequency” that is indicative of the propensity for the two primer target regions to be spatially brought together by chromatin-associating factors.

The 3C assays indeed show that, after crosslinking, the centromeric primers oXD159 and oXD162 could amplify with primers hybridizing to Mcd1p peaks that were 3 to 15 kb away (*e.g.*, oXD159 + CEN1L 5kb or CEN1R 5kb, and oXD162 + CEN16L 8kb or CEN16R 3kb; Figure 6B). Some of the amplification products spanned a region with a conspicuous Mcd1p signal without Sgo1p (*e.g.*, oXD159 + CEN1L 20kb, oXD162 + CEN16L 15kb), consistent with the idea that chromosomal loops generated by the cohesin complex is upstream to and a prerequisite for Sgo1p localization (Stephens *et al.* 2011; Verzijlbergen *et al.* 2014). The crosslinking frequency from G2/M nuclei was in general higher than G1 (orange *vs.* blue bars), which indicates that the nuclear architecture climaxes during mitosis, but may be partially preserved after exiting from M phase. This notion is consistent with the weak but readily recognizable Mcd1p peaks in cells arrested at G1 (Figure 5).

## Discussion

This work captures high-resolution genome-wide localization of Sgo1p in mitotic *S. cerevisiae* cells. On each chromosome, Sgo1p displays a tripartite localization domain consisting of a middle centromeric and typically two flanking pericentric peaks. Some chromosomes have a few extra peaks (*e.g.*, Chr.V) while the PC peaks of several others (*e.g.*, Chr. XII and XIII) are weaker. Presently, it is unclear what contributes to such differentiation. Because Sgo1p co-localizes with the cohesin complex, and that cohesin localization is tied to transcription (Lengronne *et al.*, 2004), we suspect that differential Sgo1p intensities may be related to the transcription status of the local genes. Regardless of the reason, it is very clear that Sgo1p is restricted to regions enriched for cohesin. Despite that cohesin is recruited to numerous loci across the genome, Sgo1p only rendezvouses with the centromeric and the adjacent pericentric cohesin. This confined localization of Sgo1p requires an intact tension sensing motif of histone H3. Ectopic transcription that disrupts pericentric cohesin localization also dislodges Sgo1p *in situ*. Overexpression causes Sgo1p to expand its presence, but the new Sgo1p peaks have high propensity to co-localize with cohesin. This unique trident shape of Sgo1p domain on each chromosome appears to be associated with chromatin looping in mitosis, thus linking higher-ordered chromatin architecture to positioning Sgo1p for the crucial tension sensing function of segregation.

Studies of yeast and human cells have demonstrated the importance of cohesin in Sgo1p recruitment to pericentromere (Kiburz *et al.* 2005; Liu *et al.* 2013a). However, cohesin alone is not sufficient for the pericentric retention of Sgo1p. The tension sensing motif of H3 is also required for keeping Sgo1p in this region to ensure error-free segregation. While a Gly-to-Ser mutation in the TSM has no effect on cohesin localization, both pericentric and centromeric (though to a lesser extent) enrichment of Sgo1p is compromised ((Luo *et al.* 2010) and Figure 3C). The establishment of the centromeric and pericentric domain of Sgo1p likely follows a spillover model in that Sgo1p is first recruited to the centromeres via direct association with Cse4p (Mishra *et al.* 2017) and histone H2A phosphorylated at Ser121 by kinase Bub1p (Fernius and Hardwick 2007; Kawashima *et al.* 2010). Congregation of Sgo1p molecules at centromeres permits its spread to the adjacent pericentric nucleosomes where cohesin has already been loaded. This spread may result from the turnover of a transient complex involving Sgo1p and centromeric proteins. Alternatively, the homodimerization activity of Sgo1p, evidenced by yeast two-hybrid tests (Mishra *et al.* 2017), may facilitate the growth of the Sgo1p domain from centromeres to pericentric regions where the cohesin complex resides. By binding to nucleosomes, cohesin may also help to make the tension sensing motif more accessible for Sgo1p before biorientation is established (Fernius and Hardwick 2007; Kawashima *et al.* 2010; Luo *et al.* 2010; Luo *et al.* 2016). Due possibly to the total pool size of Sgo1p, it only spreads to the first and nearest cohesin cluster. Overexpression of Sgo1p can further its spread primarily to adjacent pre-existing cohesin conglomerates (Figure 2).

The distinct kinetics of engaging Sgo1p and cohesin (Mcd1p) at *CEN16* (Figure 5) and *CEN1* (Figure S5) is consistent with the notion that cohesin organizes chromatin into a platform for mitotic machinery to execute error-free segregation. Mcd1p appears earlier than Sgo1p but fluctuates in abundance before metaphase. In the meantime, Sgo1p continues to accumulate at CEN and PC peaks until it reaches the maximum. When cells enter anaphase, Sgo1p dissipates. It is critical that before Mcd1p levels climb to the highest, Sgo1p already starts disappearing from CEN and PC regions (compare T_60’_ and T_75’_, Figure 5 and Figure S5). This time difference echoes the report of tension-dependent removal of Sgo1p from chromatin at the juncture of metaphase and anaphase (Nerusheva *et al.* 2014), and is consistent with the model that the removal of Sgo1p from chromatin is registered by cells as achieving biorientation.

The centromeric and pericentric clusters of Sgo1p appear almost simultaneously, leaving the intervening areas relatively free of Sgo1p throughout the lifespan of these peaks. The ChIP-qPCR data in Figure 5 were obtained from synchronous cells collected every 7.5 min in the M phase. Despite the short intervals for sampling, it is formally possible that an exceedingly fast mechanism pushes Sgo1p along the nucleosomes from CEN to PC. A high-precision, single-cell or fast-kinetics approach may provide a definitive answer. Results from the current resolution favor the hopping model for the establishment of PC peaks of Sgo1p.

Considering that the histone H3 tension sensing motif decorates the whole genome and functions without a post-translational modification, the non-continuous nature of the confined Sgo1p peaks on each chromosome strongly suggests physical hindrance in these Sgo1p-free intervening sections. Our recent findings that Gcn5p acts as a negative regulator for tension sensing motif and Sgo1p functional interaction (Luo *et al.* 2016; Buehl *et al.* 2018) alludes to an intriguing possibility that Gcn5p, acetylated H3, or a downstream effector may prevent Sgo1p from binding to the chromosome arms. ChIP-seq data show a lack of correlation between Gcn5p and these Sgo1p-free valleys in mitosis (Figure 3), arguing against a direct, physical role of Gcn5p. Rather, we favor the possibility that a structural feature dictates the accessibility of pericentric chromatin to Sgo1p. Indeed, the chromosome conformation capture results (Figure 6) show that the DNA around the centromere loops into a higher-ordered structure that includes centromere and the adjacent Sgo1p and cohesin clusters, a scenario reminiscent of the C-loop model put forth by Bloom and colleagues (Yeh *et al.* 2008; Salmon and Bloom 2017). The C-loop conformation posits that pericentric chromatin harbors alternating cohesin and condensin complex clusters. Condensin and the associated chromatin in pericentromere are restricted to the microtubule axis between spindle pole bodies, whereas cohesin and the cognate CARs are radially positioned, forming the wall of a barrel. In this model, multiple layers of chromatin loops distribute axially, with the top and bottom of this barrel being the clustered centromeres from all 16 chromosomes. Poleward pulling from biorientation stretches the length of this barrel and narrows its diameter.

**Figure 7:**
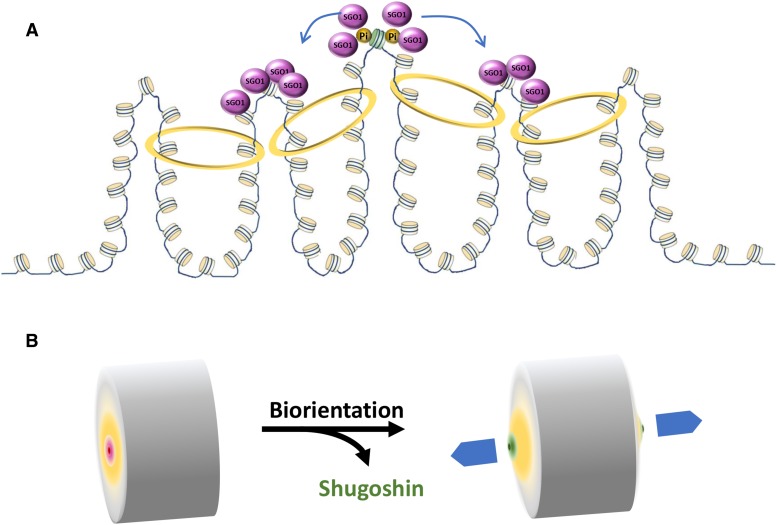
Model for the formation and dynamics of Sgo1p chromatin domain. A. Sgo1p is first recruited to the centromeres via association with phosphorylated histone H2A (Pi). Centromere-bound Sgo1p then spreads to the nearby cohesin-occupied region. B. At the whole genome level, congregation of centromeres aligns the adjacent chromatin loops to form concentric rings (gradient yellow circle) that become the two terminals of the chromatin column. Prior to biorientation, Sgo1p (gradient red circle) resides on the centromere cluster and the first ring of chromatin loops. Poleward pulling from bipolar attachment stretches the centromeric and pericentric chromatin, resulting in a conformational change (gradient green circle) and evicting Sgo1p.

How does Sgo1p fit into the tension sensing function? Taking together the ChIP-seq and 3C results, we suggest that cohesin is responsible for creating and joining multiple loops in pericentromere. With centromeres clustering in the center (Jin *et al.* 1998), these cohesin-capped loops (Figure 7A) can be viewed as a series of concentric circles (Figure 7B). Sgo1p is recruited to the centromere cluster, from which it encroaches radially to the first pericentric cohesin circle (red circles, Figure 7B). Biorientation instigates both intra- and inter-chromosomal tension (Salmon and Bloom 2017). The increased space between individual nucleosomes causes a conformational change of the tension sensing motif (Luo *et al.* 2010; Luo *et al.* 2016) or even nucleosome dissociation from pericentromere (Lawrimore *et al.* 2015). In either case, Sgo1p loses its footings and dissipates from chromatin (Figure 7B, green circles). Tension-induced clearance of Sgo1p in pericentromere signals biorientation to the spindle assembly checkpoint (Nerusheva *et al.* 2014). Anaphase thus ensues. This model provides a mechanistic explanation for the mitotic delay caused by Sgo1p overexpression (Clift *et al.* 2009). Biochemical fractionation experiments demonstrated that yeast cells do not seem to have a soluble pool of Sgo1p, but rather keep all Sgo1p molecules in the CEN/PC region (Buehl *et al.* 2018). If true, the overall size of the Sgo1p motif on chromosomes (red circles, Figure 7B) would be dictated by the number of Sgo1p molecules. Overexpression raises Sgo1p levels and expands the range of Sgo1p occupancy to the next cohesin circle farther from the centromere cluster. Consequently, more extended axial separation of kinetochores is required in order to evict the outermost Sgo1p molecules. Assuming that the quantitative removal of Sgo1p from centromeric and pericentric regions signals biorientation, Sgo1p overdose would require more time to clear Sgo1p before anaphase onset, resulting in mitotic delay. On the contrary, deleting Sgo1p or preventing the formation of the pericentric Sgo1p domain by mutating the tension sensing motif would be interpreted erroneously as biorientation by cells, thus triggering precocious anaphase onset and aneuploidy (Indjeian *et al.* 2005; Luo *et al.* 2010).
